# Convergent and discriminative validity of the Frail-VIG index with the EQ-5D-3L in people cared for in primary health care

**DOI:** 10.1186/s12877-021-02186-x

**Published:** 2021-04-13

**Authors:** Juan-José Zamora-Sánchez, Edurne Zabaleta-del-Olmo, Vicente Gea-Caballero, Iván Julián-Rochina, Gemma Pérez-Tortajada, Jordi Amblàs-Novellas

**Affiliations:** 1 Gerència Territorial de Barcelona, Institut Català de la Salut, Gran Via Corts Catalanes 587 àtic, 08007 Barcelona, Spain; 2 School of Nursing, Universitat de Barcelona, Barcelona, Spain; 3 Fundació Institut Universitari per a la recerca a l’Atenció Primària de Salut Jordi Gol i Gurina (IDIAPJGol), Barcelona, Spain; 4 Universitat Autònoma de Barcelona, Bellaterra, Cerdanyola del Vallès, Spain; 5 Nursing Department, Faculty of Nursing, Universitat de Girona, Girona, Spain; 6 Nursing school “La Fe”, Valencia, Spain; 7 GREIACC Research Group, Instituto de Investigación Sanitaria La Fe, Valencia, Spain; 8 Nursing Department, Universitat de Valencia, Valencia, Spain; 9 Fragilidad y Deterioro Cognitivo (FROG) Research Group, Universitat de Valencia, Valencia, Spain; 10 Primary care centre “Fondo”, Gerència Territorial Metropolitana Nord, Institut Català de la Salut, Santa Coloma de Gramenet, Spain; 11 Central Catalonia Chronicity Research Group (C3RG), Centre for Health and Social Care Research (CESS), Universitat de Vic – University of Vic-Central University of Catalonia (UVIC-UCC), 08500 Vic, Spain

**Keywords:** Frailty, Health status, Primary health care, Psychometrics, Validation studies as topic

## Abstract

**Background:**

The Frail-VIG frailty index has been developed recently. It is an instrument with a multidimensional approach and a pragmatic purpose that allows rapid and efficient assessment of the degree of frailty in the context of clinical practice. Our aim was to investigate the convergent and discriminative validity of the Frail-VIG frailty index with regard to EQ-5D-3L value.

**Methods:**

We carried out a cross-sectional study in two Primary Health Care (PHC) centres of the Catalan Institute of Health (Institut Català de la Salut), Barcelona (Spain) from February 2017 to January 2019. Participants in the study were all people included under a home care programme during the study period. No exclusion criteria were applied. We used the EQ-5D-3L to measure Health-Related Quality of Life (HRQoL) and the Frail-VIG index to measure frailty. Trained PHC nurses administered both instruments during face-to-face assessments in a participant’s home during usual care. The relationships between both instruments were examined using Pearson’s correlation coefficient and multiple linear regression analyses.

**Results:**

Four hundred and twelve participants were included in this study. Frail-VIG score and EQ-5D-3L value were negatively correlated (*r* = − 0.510; *P* < 0.001). Non-frail people reported a substantially better HRQoL than people with moderate and severe frailty. EQ-5D-3L value declined significantly as the Frail-VIG index score increased.

**Conclusions:**

Frail-VIG index demonstrated a convergent validity with the EQ-5D-3L value. Its discriminative validity was optimal, as their scores showed an excellent capacity to differentiate between people with better and worse HRQoL. These findings provide additional pieces of evidence for construct validity of the Frail-VIG index.

## Background

Validity is defined as “the degree to which an instrument truly measures the construct(s) it purports to measures” [1]. Validation is a continuous process and different forms of validation can be applied. Criterion validity and construct validity are two types of validity. Criterion validity is applicable when there is the gold standard for the construct that is measured, it refers to the degree to which the scores of a measurement instrument are an adequate reflection of a gold standard [1, 2]. By contrast, construct validity is applicable when there is no gold standard, it refers to the degree to which the scores of a measurement instrument are consistent with the available knowledge about the construct [1, 2].

On the other hand, frailty is defined as “a clinical state in which there is an increase in an individual’s vulnerability to develop negative health-related events (including disability, hospitalizations, institutionalizations, and death) when exposed to endogenous or exogenous stressors” [3]. It is a complex and multidimensional concept for which there are numerous and multiple operational definitions. This fact has contributed to the lack of an accepted gold standard [3, 4]. As a result, most frailty measurement instruments assess their validity by analysing the degree of consistency of their scores with different hypotheses about their relationship with other instruments or the differences between relevant groups. In other words, hypothesis testing for construct validity studies is carried out because of the difficulty in assessing criterion validity.

A new frailty index, the Frail-VIG index, has been developed recently [5, 6]. It is an instrument with a multidimensional approach and a pragmatic purpose that allows rapid and efficient assessment of the degree of frailty in the context of clinical practice. This measurement instrument has shown to have an optimal capacity to predict two-year mortality (Area Under Curve 0.85) [6]. The relationship of their scores with those of the Clinical Frailty Scale [7] has been evaluated in a cross-sectional study and a strong positive correlation (*r* = 0.706) has been established [8]. All of these studies have been conducted in an inpatient hospital setting and there have been no studies in primary health care (PHC) settings.

Previous research suggests an association between frailty and worse quality of life, but its findings are mixed and inconsistent. However, recent systematic reviews show a consistent negative association between frailty and quality of life among community-dwelling people [9, 10]. Besides, the validity of a measuring instrument does not reside in the instrument itself but in how it is used and hence depends on its appropriateness to the target population and the specific context of administration [2]. Therefore, a good approach to further developing evidence of the validity of the Frail-VIG index would mean to analyse the relationship of its scores with those of another instrument that measures the quality of life (both instruments administered in the context of PHC).

Consequently, we carried out this study in a PHC setting to investigate the convergent and discriminative validity of the Frail-VIG index regard to health-related quality of life (HRQoL) measured by the EQ-5D three-level version (EQ-5D-3L). Concerning convergent validity, we hypothesised that the relationship of scores between instruments was moderate to strong and negative. With regard to discriminative validity, we hypothesised that non-frail people would have higher scores on HRQoL than frail people.

## Methods

### Study design

We conducted a cross-sectional study on measurement properties.

### Setting and participants

The study was carried out in two PHC centres of the Catalan Institute of Health (Institut Català de la Salut), Barcelona (Spain) from February 2017 to January 2019. In these centres, people who cannot visit the centre for PHC services are included under a home care programme and are cared at-home by PHC centre’s professionals. Participants in the study were all people included under a home care program during the study period. No exclusion criteria were applied.

### Variables and data measurements

We used the EQ-5D-3L to measure HRQoL which is one of the most widely used measurement instruments [11–13]. It is a generic measurement instrument because it measures HRQoL in a way that can be used across different types of patients, health conditions, and treatments. This instrument comprises two parts. The first part consists of five dimensions: mobility, self-care, usual activities, pain/discomfort, and anxiety/depression. Each dimension has three levels: no problems, some problems, and extreme problems. Unique health status is defined by combining a level of each of the five dimensions resulting in a five-digit number, with 11,111 reflecting the best possible health status and 33,333 the worst. The second part of the instrument is the EQ VAS which comprises a visual analogue scale from 0 (the worst health imaginable) to 100 (the best health imaginable). EQ-5D-3L is designed for self-completion by respondents, but several other modes of administration (interview administered, face-to-face interview, or telephone interview) are also possible. Existing research has established that self-completion and assisted completion produce equivalent scores overall and therefore both methods can be used [14, 15]. For the present study we used the Spanish face-to-face interview version as a large majority of the participants were unable to read and write [16].

Frailty was measured using the original Spanish version of Frail-VIG index. It is composed of 22 items that evaluate 25 deficits based on the comprehensive geriatric assessment [5, 6]. It is constructed using only variables recorded during the usual clinical evaluation process. The value of the index is obtained from the sum of the identified deficits divided by 25, the total number of potential deficits, so the higher the presence of deficits the higher the score in the index. Likewise, different index cut-off points have been established that distinguish between four degrees of frailty: non-frailty, < 0.20; mild, 0.20 to 0.35; moderate, 0.36 to 0.50 and severe > 0.50 [6].

Researchers developed an instruction manual for the administration of instruments. Trained PHC nurses administered both instruments during face-to-face assessments in a participant’s home during usual care. These interviews had an average duration of 30 min. A pilot test with 20 participants was also carried out to detect possible problems and to introduce improvement strategies. After this pilot test, no changes in the procedure were necessary.

### Statistical methods

We applied a scoring algorithm based on the Spanish population to convert EQ-5D-3L states into a single summary value [13, 17]. This value is “attached to an EQ-5D profile according to a set of weights that reflect, on average, people’s preferences about how good or bad the state is” [18] and ranges from 1 (full health) to 0 (a state as bad as being death), although there are negative values for the value, corresponding to those states of health that are rated as worse than death. This value is often used in economic evaluations, but it can also be used to describe the health of a population or the severity of disease among patients [19].

We calculated central tendency and dispersion measures for the quantitative variables. For categorical variables, we estimate absolute and relative frequencies. The relationships between the Frail-VIG index and the EQ-5D-3L value were examined using Pearson’s correlation coefficient and multiple linear regression analyses. Correlation coefficients of ≤0.29 were considered weak, 0.30–0.49 as low, 0.50–0.69 as moderate, and ≥ 0.70 was considered strong correlation [20]. To examine whether non-frail people had higher scores on HRQoL than frail people, a one-way ANOVA was conducted, with frailty status as the independent variable and the EQ-5D-3L value as the dependent variable. We used the statistical software IBM SPSS Statistics version 24 for all analyses.

## Results

Four hundred and twelve participants were included in this study. Table 1 shows their general characteristics and frailty status according to the Frail-VIG index scores.

**Table 1:** General characteristics of participants overall and by frailty states. Values are absolute frequencies (percentages) unless stated otherwise

			Frailty states according to Frail-VIG index			
		Total (*n* = 412)	Non-frailty < 0.20 (*n* = 12)	Mild frailty 0.20–035 (*n* = 116)	Moderate frailty 0.36–050 (*n* = 191)	Severe frailty > 0.50 (*n* = 93)
**Variable**	**Description**					
Age, mean (SD)		88.0 (8.1)	86.7 (8.0)	88.8 (7.4)	88.1 (8.2)	86.8 (8.8)
Female		282 (68,4)	9 (75.0)	81 (69.8)	126 (66.0)	66 (71.0)
Health-related quality of life	EQ-5D-3L value, mean (SD)	0.30 (0.23)	0.48 (0.19)	0.42 (0.20)	0.29 (0.22)	0.13 (0.15)
**Domains and variables of Frail-VIG frailty index**						
*Functional domain*						
IADLs: Money management	Needs help managing financial matters (bank, shops, or restaurants)	350 (85.0)	5 (41.7)	79 (68.1)	173 (90.6)	93 (100.0)
IADLs: Telephone use	Needs help using the telephone	132 (32.0)	0 (0.0)	4 (3.4)	58 (30.4)	70 (75.3)
IADLs: Medication management	Needs assistance in preparing or administering medications	304 (73.8)	0 (0.0)	51 (44.0)	161 (84.3)	92 (98.9)
ADLs: Barthel index (BI)	No dependency (BI > = 95)	6 (1.5)	1 (8.3)	3 (2.6)	2 (1.0)	0 (0.0)
	Mild-moderate dependency (BI from 90 to 65)	143 (34.7)	11 (91.7)	79 (68.1)	51 (26.7)	2 (2.2)
	Moderate-severe dependency (BI from 60 to 25)	175 (42.5)	0 (0.0)	33 (28.4)	111 (58.1)	31 (33.3)
	Absolute dependency (BI <=20)	88 (21.4)	0 (0.0)	1 (0.9)	27 (14.1)	60 (64.5)
*Nutritional domain*						
Malnutrition	Weight loss > = 5% in the las 6 months	70 (17.0)	0 (0.0)	10 (8.6)	34 (17.8)	26 (28.0)
*Cognitive domain*						
Degree of cognitive impairment	No cognitive impairment	198 (48.1)	12 (100.0)	90 (77.6)	86 (45.0)	10 (10.8)
	Mild-moderate cognitive impairment (equivalent to GDS < =5)	171 (41.5)	0 (0.0)	26 (22.4)	93 (48.7)	52 (55.9)
	Severe-very severe cognitive impairment (equivalent to GDS > =6)	43 (10.4)	0 (0.0)	0 (0.0)	12 (6.3)	31 (33.3)
*Emotional domain*						
Depressive syndrome	Need for antidepressant medication	158 (38.3)	0 (0.0)	25 (21.6)	82 (42.9)	51 (54.8)
Insomnia/anxiety	Frequent need for benzodiazepines or other psychiatric drugs with a sedative effect for insomnia/anxiety	226 (54.9)	4 (33.3)	55 (47.4)	104 (54.5)	63 (67.7)
*Social domain*						
Social vulnerability	Do health care professionals perceive the presence of social vulnerability?	212 (51.5)	4 (33.3)	69 (59.5)	93 (48.7)	46 (49.5)
*Geriatric syndromes*						
Delirium	Presence of delirium and/or behaviour disorder requiring antipsychotic drugs in the last6 months	119 (28.9)	0 (0.0)	8 (6.9)	48 (25.1)	63 (67.7)
Falls	In the last 6 months, ≥2 falls or hospitalization due to a fall.	112 (27.2)	0 (0.0)	21 (18.1)	56 (29.3)	35 (37.6)
Ulcers	Presence of ulcer (pressure or vascular, any grade)	85 (20.6)	0 (0.0)	9 (7.8)	39 (20.4)	37 (39.8)
Polypharmacy	Taking ≥5 drugs	365 (88.6)	8 (66.7)	91 (78.4)	178 (93.2)	88 (94.6)
Dysphagia	Difficulty swallowing when eating or drinking? Presence of aspiration respiratory infections during the last 6 months?	75 (18.2)	0 (0.0)	2 (1.7)	24 (12.6)	49 (52.7)
*Severe symptoms*						
Pain	Need for ≥2 conventional analgesics and/or strong opioids for pain control	87 (21.1)	3 (25.0)	15 (12.9)	49 (25.7)	20 (21.5)
Dyspnoea	Basal dyspnoea impeding the ability to leave the house and/or opioids are frequently needed	19 (4.6)	0 (0.0)	3 (2.6)	10 (5.2)	6 (6.5)
*Diseases*						
Cancer	Active cancer	36 (8.7)	0 (0.0)	4 (3.4)	24 (12.6)	12 (12.9)
Respiratory	Presence of any type of chronic respiratory disease (COPD, restrictive lung disease...)	116 (28.2)	1 (8.3)	23 (19.8)	61 (31.9)	31 (33.3)
Cardiac	Presence of any type of chronic heart disease (heart failure, ischemic cardiomyopathy, arrhythmia	248 (60.2)	2 (16.7)	57 (49.1)	124 (64.9)	65 (69.9)
Neurological	Presence of any type of neurodegenerative disease (Parkinson, ALS...) or a history of stroke (ischemic or haemorrhagic)	151 (36.7)	0 (0.0)	24 (20.7)	75 (39.3)	52 (55.9)
Digestive	Presence of any type of chronic digestive disease (chronic liver disease, cirrhosis, chronic pancreatitis, inflammatory bowel disease, ...	39 (9.5)	1 (8.3)	8 (6.9)	22 (11.5)	8 (8.6)
Renal	Presence of chronic renal failure (GFR < 60)	204 (49.5)	4 (33.3)	57 (49.1)	102 (53.4)	41 (44.1)

*ADLs* Activities of Daily Living, *ALS* Amyotrophic lateral sclerosis, *COPD* Chronic Obstructive Pulmonary Disease, *GDS* Global deterioration scale, *GFR* Glomerular Filtration Rate, *IAVDs* Instrumental Activities of Daily Living, *SD* Standard deviation

Table 2 describes the 10 most frequent EQ-ED-3 L profiles according to frailty status. The worst of these profiles was most prevalent among people with severe frailty, while the best was more frequent among non-frailty people.

**Table 2:** Prevalence of the 10 most frequently observed EQ-ED-3 L profiles and frequency of reporting of the worst possible profile according to frailty state (these were the 10 profiles of 63.1% (*n* = 260) of the study population (the most frequent profiles in each of the frailty states are highlighted in bold).

Profiles, n (%)	Total (*n* = 412)	Frailty states according to Frail-VIG index			
		Non-frailty < 0.20 (*n* = 12)	Mild frailty 0.20–035 (*n* = 116)	Moderate frailty 0.36–050 (*n* = 191)	Severe frailty > 0.50 (*n* = 93)
*Top 10 profiles*					
33322	62 (15.0)	0 (0.0)	4 (3.4)	24 (12.6)	**34 (36.6)**
22222	60 (14.6)	1 (8.3)	**27 (23.3)**	**26 (13.6)**	6 (6.5)
23322	29 (7.0)	0 (0.0)	2 (1.7)	18 (9.4)	9 (9.7)
22221	23 (5.6)	1 (8.3)	12 (10.3)	9 (4.7)	1 (1.1)
22211	22 (5.3)	1 (8.3)	7 (6.0)	14 (7.3)	0 (0.0)
22212	20 (4.9)	1 (8.3)	6 (5.2)	10 (5.2)	3 (3.2)
33311	13 (3.2)	0 (0.0)	0 (0.0)	9 (4.7)	4 (4.3)
22322	12 (2.9)	0 (0.0)	6 (5.2)	6 (3.1)	0 (0.0)
33321	12 (2.9)	0 (0.0)	0 (0.0)	3 (1.6)	9 (9.7)
21221	7 (1.7)	**2 (16.7)**	4 (3.4)	1 (0.5)	0 (0.0)
*Worst possible*					
33333	4 (1.0)	0 (0.0)	0 (0.0)	2 (1.0)	2 (2.2)

Each digit of profile label corresponds to one of the five dimensions of the EQ-5D-3L (from left to right): Mobility, Self-Care, Usual activities, Pain and discomfort, and Anxiety and depression

Level 1, no problems; Level 2, moderate problems, and level 3, extreme problems

Pearson’s correlation coefficient between Frail-VIG index and EQ-5D-3L value was negative and moderate (*r* = −0.510; *P* < 0.001). After adjusting for age and sex variables, the multiple linear regression model revealed that Frail-VIG index independently correlated with EQ-5D-3L value (B = -0.945; 95% Confidence Interval, −1.098 to − 0.791; R^2^ = 0.287). As you can see from Table 1, non-frail people reported a substantially better HRQoL than people with moderate and severe frailty people. Likewise, closer inspection of Table 3 shows that the EQ-5D-3L value declined significantly as the Frail-VIG index score increased.

**Table 3:** EQ-5D-3L value (from “0”, “a state as bad as being dead” to “1”,“full health”) for the total study population and the seven groups based on their Frail-VIG score (from “0”, “absence of frailty” to “1”, “severe frailty”)

		EQ-5D-3L value	
Frail-VIG index score	n	Mean (SD)	Median (25th–75th)
0–0.15	4	0.55 (0.21)	0.62 (0.32–0.69)
0.16–0.25	50	0.49 (0.17)	0.54 (0.32–0.60)
0.26–0.35	74	0.38 (0.20)	0.49 (0.22–0.54)
0.36–0.45	150	0.31 (0.23)	0.23 (0.12–0.54)
0.46–0.55	72	0.21 (0.20)	0.12 (0.04–0.33)
0.56–065	55	0.12 (0.13)	0.09 (0.04–0.15)
0.66–1	7	0.05 (0.07)	0.04 (0.04–0.12)
Total	412	0.30 (0.23)	0.23 (0.09–0.49)

ANOVA (F = 22.887; degrees of freedom = 6; *P* < 0.001

EQ-5D-3L value ranged from − 0.08 to 1

## Discussion

In this study, involving people cared at-home by PHC professionals, we found that the Frail-VIG index demonstrated convergent validity with the EQ-5D-3L value. Furthermore, its discriminative validity was optimal, as their scores showed an excellent capacity to differentiate between people with better and worse HRQoL.

Consistent with the literature, this research found that HRQoL and frailty were negatively associated among community-dwelling older people [9, 10]. Studies on other cumulative deficit models of frailty have also found a negative relationship between the frailty state and the scores of instruments measuring HRQoL, well-being and life satisfaction [9, 10, 21–23]. However, the results of this study show that not only is the frailty state associated with a worse HRQoL but also that there is a linear association between both indexes (Frail-VIG index and EQ-5D-3L value). Hypothesis testing is an ongoing process, so the more hypotheses are tested the more evidence is generated for construct validity [24]. Therefore, this research supports evidence from previous studies carried out in the hospital setting on the construct validity of the Frail-VIG index [6, 8].

This study has several strengths. Most validation studies of measurement instruments of frailty in community-dwelling people focus on demonstrating their predictive potential for adverse outcomes or resource use, such as disability, institutionalisation or hospital admissions [25–27]. In contrast, studies that analyse their relationship to more positive outcomes such as HRQoL are less common [10, 28]. Likewise, the use of the Frail-VIG index in primary care has been poorly studied, and this is one of the first studies to analyse its construct validity in this care setting. However, some limitations exist. The representativeness of non-frailty people is very low (12 people), probably because the study population were people in a home-care programme. A greater representation of this population might have influenced the Frail-VIG index’s discriminative validity observed. In addition, we used the EQ-5D values for this assessment of the measurement properties of the Frail-VIG index instead of other values such as the EQ VAS. The EQ VAS can be considered a measure closer to the patient’s perspective than the EQ-5D values [29]. Nevertheless, as Devlin and Parkin point out [19], being able to summarise and represent a health profile with a single value has important advantages, including simplifying statistical analyses. On the other hand, both the EQ-5D values and the EQ VAS are able to discriminate between the quality of life of most groups of individuals with different socio-demographic factors, and those with or without clinical conditions [30]. Furthermore, some studies [31] and experts in the field of psychometrics [32] report that older people experience difficulties in understanding and completing direct estimation methods, such as the visual analogue scale. For all these reasons, we chose the EQ-5D values to carry out this psychometric study.

People living with frailty risk experiencing a decline in their quality of life [9, 10, 33]. The findings of this study suggest that the interventions aimed at decreasing frailty could have the added benefit of improving the HRQoL. PHC professionals are naturally positioned to identify frailty early and to implement interventions that prevent related adverse effects on the most vulnerable people [4]. Moreover, the assessment of frailty in PHC settings requires tools that are not time consuming as well as valid and reliable which is way the Frail-VIG index could provide a useful and appropriate tool for this care setting [34].

## Conclusions

This study has identified a negative moderate correlation between the Frail-VIG index and the EQ-5D-3L values. It has also shown that the Frail-VIG index was able to discriminate significantly home-dwelling older people according to their HRQoL. These findings provide additional pieces of evidence for construct validity of the Frail-VIG index. Further research is needed on this new measurement instrument to determine its suitability for screening and preventing adverse effects of frailty in PHC settings.

## Data Availability

The data that support the findings of this study are available from the corresponding author, [EZO], upon reasonable request.

## Abbreviations

ADLs: Activities of Daily Living
ALS: Amyotrophic Lateral Sclerosis
COPD: Chronic Obstructive Pulmonary Disease
GDS: Global Deterioration Scale
GFR: Glomerular Filtration Rate
HRQoL: Health Related Quality of Life
IAVDs: Instrumental Activities of Daily Living
PHC: Primary Health Care
SD: Standard Deviation
