# Correction to: Convergent and discriminative validity of the Frail-VIG index with the EQ-5D-3L in people cared for in primary health care

**DOI:** 10.1186/s12877-021-02369-6

**Published:** 2021-07-12

**Authors:** Juan-José Zamora-Sánchez, Edurne Zabaleta-del-Olmo, Vicente Gea-Caballero, Iván Julián-Rochina, Gemma Pérez-Tortajada, Jordi Amblàs-Novellas

**Affiliations:** 1 Gerència Territorial de Barcelona, Institut Català de la Salut, Gran Via Corts Catalanes 587 àtic, 08007 Barcelona, Spain; 2 School of Nursing, Universitat de Barcelona, Barcelona, Spain; 3 Fundació Institut Universitari per a la recerca a l’Atenció Primària de Salut Jordi Gol i Gurina (IDIAPJGol), Barcelona, Spain; 4 Universitat Autònoma de Barcelona, Bellaterra, Cerdanyola del Vallès, Spain; 5 Nursing Department, Faculty of Nursing, Universitat de Girona, Girona, Spain; 6 Nursing school “La Fe”, Valencia, Spain; 7 GREIACC Research Group, Instituto de Investigación Sanitaria La Fe, Valencia, Spain; 8 Nursing Department, Universitat de Valencia, Valencia, Spain; 9 Fragilidad y Deterioro Cognitivo (FROG) Research Group, Universitat de Valencia, Valencia, Spain; 10 Primary care centre “Fondo”, Gerència Territorial Metropolitana Nord, Institut Català de la Salut, Santa Coloma de Gramenet, Spain; 11 Central Catalonia Chronicity Research Group (C3RG), Centre for Health and Social Care Research (CESS), Universitat de Vic – University of Vic-Central University of Catalonia (UVIC-UCC), 08500 Vic, Spain

**Correction to: BMC Geriatr 21, 243 (2021)**

**https://doi.org/10.1186/s12877-021-02186-x**

In the original publication [1] there was an incorrect funding acknowledgement. In this correction article the correct and incorrect funding acknowledgement are published. The updated information is shown in **bold**. The original article has been updated.

**Incorrect funding**

- This research was supported by the Health Department grant number (SLT008/18/00011) from the Generalitat of Catalunya (Spain) and by a grant from the Territorial Management of Barcelona of the Institut Català de la Salut (Catalan Institute of Health) to the first author (JJZS) in the 2018 edition. The funders had no role in review design, decision to publish, or preparation of this manuscript.

**Correct funding**

- This research was supported by, the Health Department grant number (SLT008/18/00011) from the Generalitat of Catalunya (Spain), by a grant from the Territorial Management of Barcelona of the Institut Català de la Salut (Catalan Institute of Health) to the first author (JJZS) in the 2018 edition, and by the MUTUAMCONVIURE Foundation's Research Award in Social and Health Care (18th Edition). The funders had no role in review design, decision to publish, or preparation of this manuscript.
